# Predicting Cell Wall Lytic Enzymes Using Combined Features

**DOI:** 10.3389/fbioe.2020.627335

**Published:** 2021-01-06

**Authors:** Xiao-Yang Jing, Feng-Min Li

**Affiliations:** College of Science, Inner Mongolia Agricultural University, Hohhot, China

**Keywords:** cell wall lytic enzymes, optimized combination feature, synthetic minority over-sampling technique, F-score, support vector machine, jackknife test

## Abstract

Due to the overuse of antibiotics, people are worried that existing antibiotics will become ineffective against pathogens with the rapid rise of antibiotic-resistant strains. The use of cell wall lytic enzymes to destroy bacteria has become a viable alternative to avoid the crisis of antimicrobial resistance. In this paper, an improved method for cell wall lytic enzymes prediction was proposed and the amino acid composition (AAC), the dipeptide composition (DC), the position-specific score matrix auto-covariance (PSSM-AC), and the auto-covariance average chemical shift (acACS) were selected to predict the cell wall lytic enzymes with support vector machine (SVM). In order to overcome the imbalanced data classification problems and remove redundant or irrelevant features, the synthetic minority over-sampling technique (SMOTE) was used to balance the dataset. The F-score was used to select features. The S_*n*_, S_*p*_, MCC, and Acc were 99.35%, 99.02%, 0.98, and 99.19% with jackknife test using the optimized combination feature AAC+DC+acACS+PSSM-AC. The S_*n*_, S_*p*_, MCC, and Acc of cell wall lytic enzymes in our predictive model were higher than those in existing methods. This improved method may be helpful for protein function prediction.

## Introduction

Bacteria are constantly around us, and bacterial infections have become a major public health problem. The overuse of antibiotics leads to the rapid rise of antibiotic-resistant strains, and people are worried that existing antibiotics will become ineffective against pathogens. Using cell wall lytic enzymes to destroy bacteria has become a viable alternative method to avoid the crisis of antimicrobial resistance (Sommer et al., 2017; Wu et al., 2017; Bhagwat et al., 2019; Cheng et al., 2020). Cell wall lytic enzymes are divided into two enzymes: endolysin and autolysin. Endolysins are phage-encoded enzymes that have evolved to degrade the bacterial cell wall (Shavrina et al., 2016). Many studies have shown that endolysin has an excellent bactericidal effect on *Staphylococcus aureus* (Ajuebor et al., 2016), *Escherichia coli* (Yan et al., 2019), *Streptococcus suis* (Der Ploeg, 2008), and other pathogens. Compared with conventional antibiotics, endolysin has many advantages, such as rapid host killing, host specificity, low chances of developing drug resistance, and efficacy against multidrug-resistant bacteria (Gondil et al., 2020). Autolysin is the other cell wall lytic enzyme that degrades some bonds in the peptidoglycan backbone of the bacterial cell wall (Usobiaga et al., 1996), and it is closely related to the life of cells and participates in the control of cell growth, cell lysis, daughter-cell separation, and biofilm formation (Kalali et al., 2019). Cell wall lytic enzymes have become a valuable tool for biological researchers in the medical and food industry and in agricultural applications (Yu, 1997).

Experimental determination of the cell wall lytic enzymes is time-consuming and laborious, so it is necessary to use an effective method to predict cell wall lytic enzymes. Recently some computational methods for predicting cell wall lytic enzymes have been proposed. Ding et al. (2009) used Chou’s amphiphilic pseudo to predict cell wall lytic enzymes; the predictive accuracy was 80.40% with jackknife test. Chen et al. (2016) developed a predictor called “Lypred” that used pseudo amino acid composition (PseAAC) as a feature vector; the predictive accuracy was 91.3% with fivefold cross-validation. Meng et al. (2020) developed a predictor called “CWLy-SVM” that employed the 473-dimensional sequence-based feature descriptor to predict cell wall lytic enzymes; the result was 95.50% with jackknife test. In this paper, the amino acid composition (AAC), the dipeptide composition (DC), the position-specific score matrix auto-covariance (PSSM-AC), and the Auto-covariance average chemical shift (acACS) were used to predict the cell wall lytic enzymes with the same datasets as investigated by Chen et al. (2016).

Data imbalance is always considered a problem in developing efficient and reliable prediction systems; in imbalanced datasets, the classifier would tend to the majority class. Here, the synthetic minority over-sampling technique (SMOTE) was used to solve the problem of imbalance. To remove redundant or irrelevant features, we selected features using the F-score algorithm. The accuracy (Acc) was 99.19% with a balanced dataset in jackknife test by using the optimized combination feature AAC+DC+PSSM-AC+acACS.

## Materials and Methods

### Benchmark Dataset

The benchmark dataset was generated by Chen et al. (2016), The dataset was taken from the Universal Protein Resource (UniProt), using the following steps to collect the sequence: (1) sequences annotated with “Inferred from homology” or “Predicted” were removed. (2) Sequences which were the fragments of other proteins were not included. (3) Sequences containing ambiguous letters such as “B,” “J,” “O,” “U,” “X,” and “Z” were excluded. To reduce homologous bias and redundancy, the program CD–HIT (Li and Godzik, 2006) was used to remove those sequences that have ≥ 40% pairwise sequence identity. Finally, 375 sequences were obtained; they contained 68 lyases and 307 non-lyases, and the dataset can be expressed as:

(1) $$S=S_{l\,y\,s\,a\,s\,e\,s}\cup S_{n\,o\,n\,l\,y\,s\,a\,s\,e\,s}$$

The dataset can be freely downloaded from http://lin-group.cn/server/Lypred/data.html.

### Feature Extraction Techniques

Feature extraction is a crucial step in developing a powerful predictor; a set of reasonable features contains more protein sequence information (Zhu et al., 2018; Yang et al., 2019; Zhang and Liu, 2019). Generally, the feature combination can boost the prediction performance. In this paper, the AAC, the DC, the PSSM-AC, and the acACS were used to predict the cell wall lytic enzymes.

#### Amino Acid Composition

The amino acid composition of proteins is the most basic feature information in all features. The protein sequence consists of 20 amino acids (A, C, D, E, F, G, H, I, K, L, M, N, P, Q, R, S, T, V, W, and Y). AAC calculates the occurrence frequency of the 20 native amino acids so that the protein sequence can be expressed as 20 features in a feature vector. It can be defined as:

(2) $$P=\left[x_{1},x_{2},x_{3},\cdots ,x_{i},\cdots ,x_{20}\right]$$

(3) $$x_{i}=\frac{n_{i}}{L}$$

Where *n*_*i*_ is the occurrence number of the 20 native amino acid in protein sequence and L is the length of the protein sequence.

#### Dipeptide Composition

Dipeptide composition (DC) is calculated as the occurrence frequency of each two adjacent amino acid residues. There are 20^∗^20 = 400 combinations of amino acid pairs. Compared with AAC, DC is a feature that considers some sequence-order information. It can be calculated as:

(4) $$P=\left[f_{1},f_{2},f_{3},\ldots ,f_{i},\ldots ,f_{400}\right]$$

(5) $$f_{i}=\frac{m_{i}}{L-1}$$

Where *m*_*i*_ is the occurrence number of i-th dipeptide in protein sequence and L is the length of the protein sequence.

#### Position-Specific Score Matrix Auto-Covariance

Position-Specific Score Matrix Auto-Covariance (PSSM-AC) is a feature that extracts the evolutionary information of a protein sequence. PSSM-AC was first proposed to predict the protein fold recognition by Dong et al. (2009). Recently, the PSSM-AC was used successfully in many works for the prediction of protein function (Zou et al., 2013; Huang and Li, 2018; Wang et al., 2019b, 2020a). In PSSM-AC, the PSI-BLAST (Position-Specific Iterative Basic Local Alignment Tool) was used to generate PSSM; the threshold of *e*-value is 0.001 and the maximum number of iterations is 3. PSSM-AC is calculated as the correlation between two residues within PSSM. This method can be represented as:

(6) $$P_{P\,S\,S\,M}=\left[\begin{array}{cccccc} R_{1,1} & R_{1,2} & \ldots & R_{1,j} & \ldots & R_{1,20}\\ R_{2,1} & R_{2,2} & \ldots & R_{2,j} & \ldots & R_{2,20}\\ \vdots & \vdots & \vdots & \vdots & \vdots & \vdots \\ R_{i,1} & R_{i,2} & \ldots & R_{i,j} & \ldots & R_{i,20}\\ \vdots & \vdots & \vdots & \vdots & \vdots & \vdots \\ R_{L,1} & R_{L,2} & \ldots & R_{L,j} & \ldots & R_{L,20} \end{array}\right]$$

(7) $$P_{P\,S\,S\,M}-A\,C\,\left(j,\mathrm{lg}\right)=\frac{1}{L-\mathrm{lg}}\,\sum_{i=1}^{L-\mathrm{lg}}\left(R_{i,j}-{\bar{R}}_{j}\right)\,\left(R_{i+\mathrm{lg},j}-{\bar{R}}_{j}\right)$$

(8) $${\bar{R}}_{j}=\frac{1}{L}\sum_{i=1}^{L}R_{i,j}\left(j=1,\ldots ,20\right)$$

Where *R*_*i,j*_ is the score of the residue of the i-th position mutated to the j-th amino acids residue in the protein sequence; a high score means a highly conserved position. L is the length of the protein sequence, *lg* is the distance along the sequence, and 0 < *lg*< L. As a result, the protein sequence generates a 20 × *lg* dimensional feature vector with PSSM-AC.

#### Auto-Covariance Average Chemical Shift

As important parameters are measured by nuclear magnetic resonance (NMR) spectroscopy, the chemical shift has been used as a powerful indicator of the protein structure. Several researchers revealed that the average chemical shift (ACS) of a particular nucleus in the protein backbone empirically correlates to its secondary structure (Sibley et al., 2003). acACS was proposed by Fan et al. (2014), In acACS, the secondary structure was converted into the average chemical shift, and then the auto-covariance function was used to construct the vector representing the protein sequence by selecting different. In this work, the secondary structure was obtained by submitting the protein sequence to PSIPRED^1^, and then the protein sequence and the corresponding secondary structure were submitted to the acACS web server^2^. It can be calculated as:

For a protein P, where each amino acid in the sequence is substituted by its averaged chemical shift, P can be expressed as:

(9) P=[A1i,A2i,A3i,…,ALi](i=N15,Cα13,Hα1,HN1)

Where ^15^*N* stands for Nitrogen, ^13^*C*_α_ for alpha Carbon, ^1^*H*_α_ for alpha Hydrogen, and ^1^*H*_*N*_ for Hydrogen linked with Nitrogen.

After we select λ = 17 and i=N15,Cα13,Hα1,H1, the acACS could be expressed as:

(10) φiλ=1L-λ∑k=1L-λ[Aki-Ak+λi](i=N15,Cα13,Hα1,HN1;λ<L)

(11) P=[φi0,φi1,φi2,…,φiλ](i=N15,Cα13,Hα1,HN1)

### Synthetic Minority Over-Sampling Technique

The numbers of non-lyases are about 4.5 times that of lyases, and this leads to imbalanced data classification problems. In order to overcome this problem, we used SMOTE to solve the problem of imbalance. SMOTE is an over-sampling approach for imbalanced data classification (Wang et al., 2018a; Zhou et al., 2019). The algorithm of SMOTE is described as follows: (1) randomly choose the samples *x*_*i*_ from the minority class, and calculate the Euclidean distance to all other samples in this class, then K nearest neighbors of this sample were selected, (2) select *x*_*i*_ samples from the k nearest neighbors, and (3) generate a new sample *x*_*new*_ by: *x*_*new*_ = *x*_*i*_ + α(*x*−*x*_*i*_), α is a random number in (0, 1). In this paper, the protein numbers of lyases and non-lyases are in equilibrium with SMOTE.

### Feature Selection

Redundant or irrelevant features will decrease the accuracy of prediction and increase computational time. In order to remove redundant or irrelevant features, a variety of feature selection techniques have been proposed: the analysis of variance (ANOVA) (Tan et al., 2018; Li et al., 2019; Zhang et al., 2020a), Max-Relevance-Max-Distance algorithms (MRMD) (Zou et al., 2016; Wan et al., 2017; Ru et al., 2019; Kwon et al., 2020), and Minimal-Redundancy-Maximal-Relevance (MRMR) (Jiao and Du, 2016; Xu et al., 2016; Wang et al., 2018b; Kabir et al., 2020) are the representative feature selection algorithms. In this study, we selected features using the F-score algorithm; the F-score algorithm was proposed by Yi-Wei (Chen and Lin, 2006). All features are ranked according to F-score values; a higher score indicates a higher likelihood that this feature is more discriminative (Zhang et al., 2020b). It can be calculated as:

(12) $$F_{i}=\frac{{\left({\bar{x}}_{i}^{\left(+\right)}-{\bar{x}}_{i}\right)}^{2}+{\left({\bar{x}}_{i}^{\left(-\right)}-{\bar{x}}_{i}\right)}^{2}}{\sum_{k=1}^{n^{+}}{\left({\bar{x}}_{k,i}^{\left(+\right)}-{\bar{x}}_{i}^{\left(+\right)}\right)}^{2}+\frac{1}{n^{-}-1}\,\sum_{k=1}^{n^{-}}{\left({\bar{x}}_{k,i}^{\left(-\right)}-{\bar{x}}_{i}^{\left(-\right)}\right)}^{2}}$$

Where ${\bar{x}}_{i}$ is the average of the i-th feature of the whole sample, ${\bar{x}}_{i}^{\left(+\right)}$ is the average of the i-th feature of the positive samples, ${\bar{x}}_{i}^{\left(-\right)}$ is the average of the i-th feature of the negative samples; *n*^+^ is the total number of positive samples, *n*^−^ is the total number of negative samples; ${\bar{x}}_{k,i}^{\left(+\right)}$ is the average of the i-th feature of the k-th sample in the positive samples, and ${\bar{x}}_{k,i}^{\left(-\right)}$ is the average of the i-th feature of the k-th sample in the negative samples.

To determine the optimal features, the incremental feature selection (IFS) (Ju and He, 2017; Tang et al., 2018) was employed based on the features ranked. The IFS procedure starts with one feature with the highest score, then adds features to the start feature based on their scores until all the features are added.

### Support Vector Machine

The support vector machine was proposed by Vapnik; the basic idea of SVM is to transform the input data into a high-dimensional Hilbert space and then determine the optional separating hyperplane. SVM has been successfully applied in the field of computational biology and bioinformatics (Fan et al., 2013; Li and Wang, 2016; Arif et al., 2018; Chen et al., 2019; Tian et al., 2019; Wang et al., 2019a; Du et al., 2020; Jing and Li, 2020; Yang et al., 2020). Therefore, we used this classifier to build our model. The radial basis function (RBF) kernel was adopted to perform prediction. The regulation parameter c and kernel width parameter γ were tuned via the grid search method. In this paper, the LibSVM package was used to predict cell wall lytic enzymes, which can be downloaded from https://www.csie.ntu.edu.tw/~cjlin/libsvm.

### Performance Evaluation

In statistical prediction, three cross-validation methods are commonly used to examine a predictor for its effectiveness in practical applications: k-fold cross-validation, independent dataset test, and jackknife test (Li and Li, 2008; Tan et al., 2019; Dao et al., 2020a,b). Among the three methods, the jackknife test is deemed the most objective and rigorous. Hence, the jackknife test was used to evaluate the performance of this paper.

In order to evaluate the predictive capability and reliability of our model, the sensitivity (Sn), specificity (Sp), Matthew’s correlation coefficient (MCC), and accuracy (Acc) (Bustamam et al., 2019; Cheng, 2019; Cheng et al., 2019; Feng et al., 2019; Malebary et al., 2019; Chen et al., 2020; Li and Gao, 2020; Wang et al., 2020b) were measured and defined by:

(13) $$s_{n}=\frac{T\,P}{T\,P+F\,N}$$

(14) $$s_{p}=\frac{T\,N}{T\,N+F\,N}$$

(15) $$M\,C\,C=\frac{T\,P\,\text{×}\,T\,N-F\,P\,\text{×}\,F\,N}{\sqrt{\left(T\,P+F\,P\right)\,\text{×}\,\left(T\,N+F\,N\right)\,\text{×}\,\left(T\,P+F\,N\right)\,\text{×}\,\left(T\,N+F\,P\right)}}$$

(16) $$A\,c\,c=\frac{T\,P+T\,N}{T\,P+T\,N+F\,P+F\,N}$$

Where TP represents the true positive, TN represents the true negative, FP represents the false positive, and FN represents the false negative.

## Results and Discussion

### The Choice of Our Model Parameters lg, and Combination Schemes of Chemical Shifts

**FIGURE 1:**
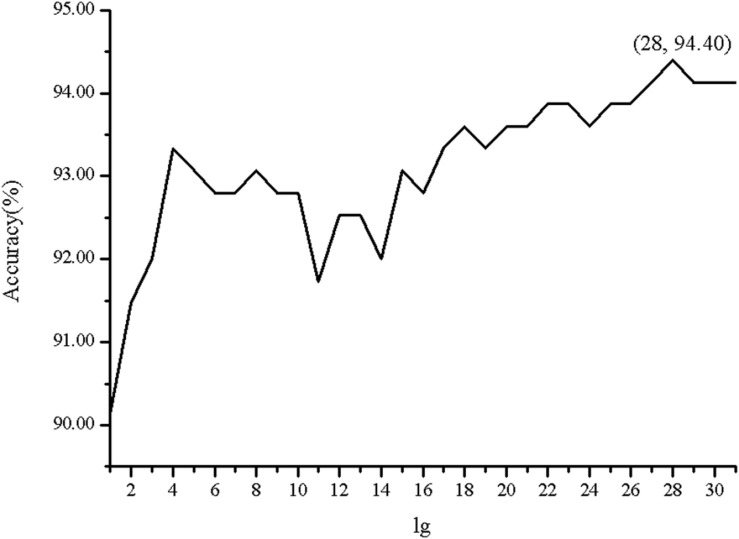
The Acc of position-specific score matrix auto-covariance (PSSM-AC) with different *lg*.

**FIGURE 2:**
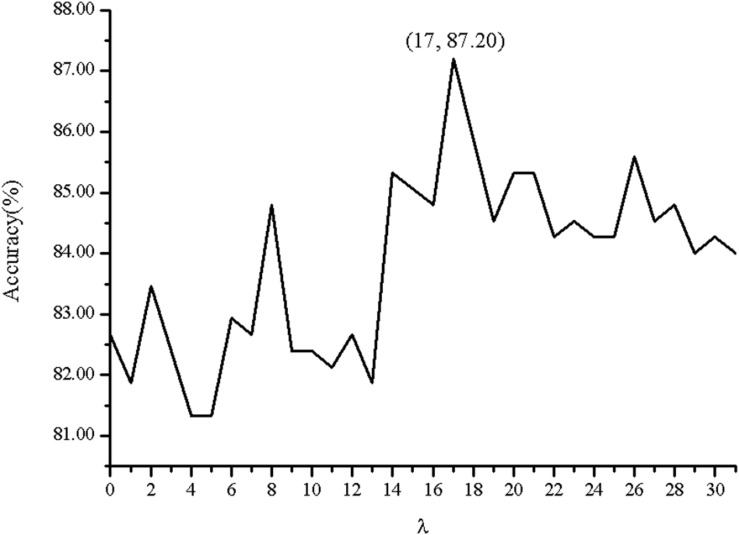
The Acc with respect to the correlation factor *λ* of the combination mode of chemically shifted atoms 15⁢N,Cα13,Hα1,H1.

**FIGURE 3:**
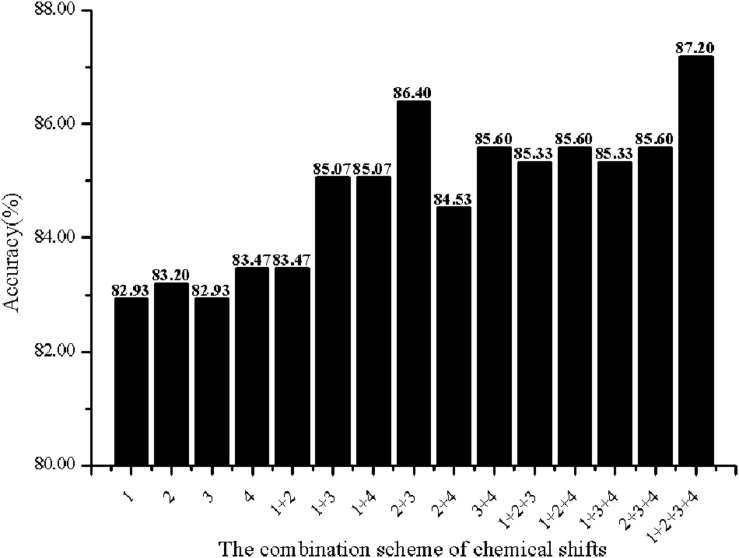
The Acc of different combination schemes of chemical shifts. Numbers denote the chemical shifts of atoms: 1 denotes ^15^*N*, 2 denotes ^13^*C*_α_, 3 denotes ^1^*H*_α_, 4 denotes ^1^*H*_*N*_.

In order to investigate the effectiveness of the predictive model, the AAC, the DC, PSSM-AC, and the auto-covariance, average chemical shift was selected to predict the cell wall lytic enzymes. Furthermore, for the sake of the best performance of predicting cell wall lytic enzyme, the lg of the distance was selected, with results in Figure 1, and the best lg was 28 when the accuracy was the highest. In addition, the combination mode of chemically shifted atoms and the best parameter λ were selected. Figure 2 shows that the best parameter λ was 17. The results of combination mode of chemically shifted atoms were shown in Figure 3; the best combination mode of chemically shifted atoms was 15⁢N,Cα13,Hα1,H1 when the accuracy was the highest.

### The Predictive Performance of Cell Wall Lytic Enzymes

**FIGURE 4:**
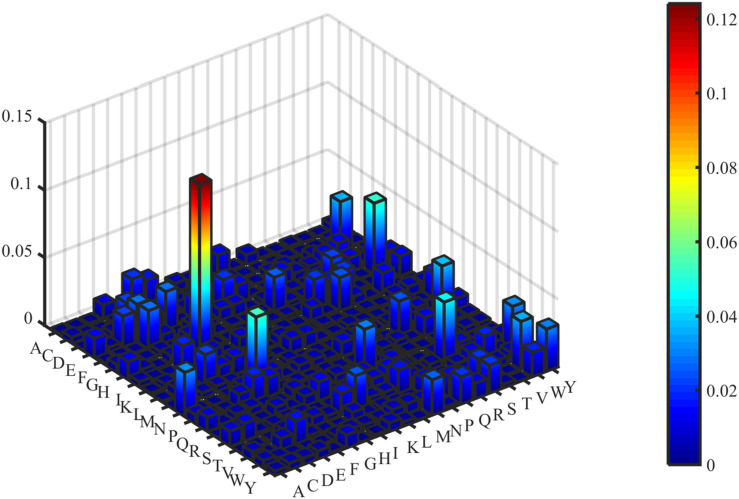
Three-dimensional heat map of DC’s F-score value.

**FIGURE 5:**
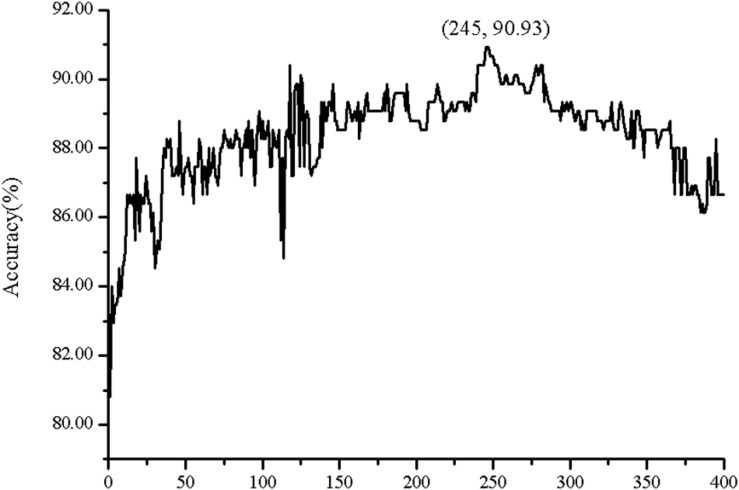
The Acc of dipeptide composition (DC) with the incremental feature selection.

**FIGURE 6:**
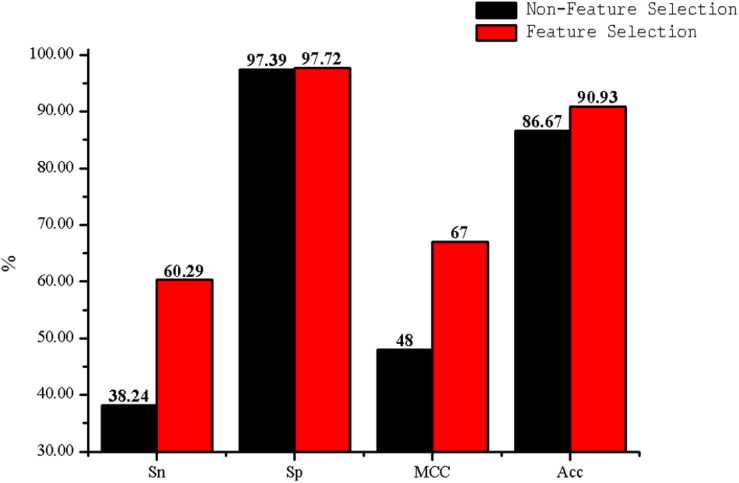
The Acc of DC with feature selection and non-feature selection.

**FIGURE 7:**
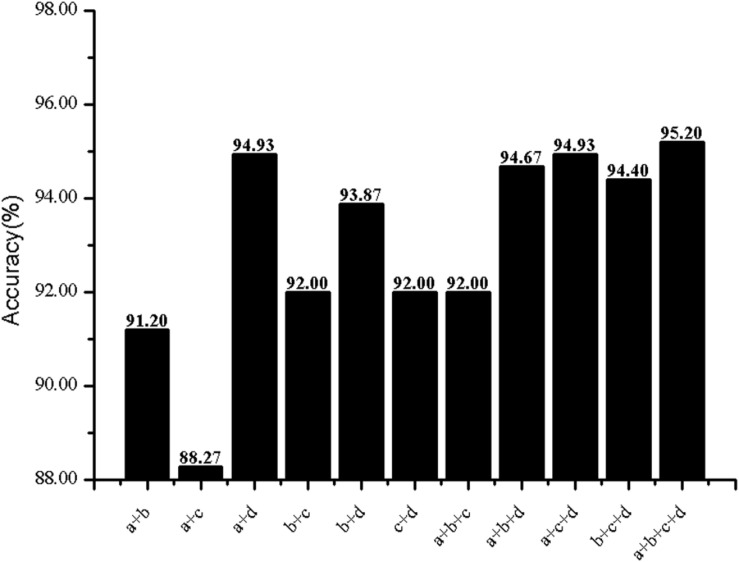
Prediction results of different combined features. Letters denote features: a for AAC, b for DC, c for acACS, d for PSSM-AC.

The predictive performance of cell wall lytic enzymes by using the SVM classification algorithm with SMOTE was listed in Table 1. The highest sensitivity (Sn), specificity (Sp), Matthew’s correlation coefficient (MCC), and accuracy (Acc) of individual parameters were 72.06%, 99.67%, 0.81, and 94.40% with jackknife test by using PSSM-AC. By comparison, the result of acACS was better than AAC and DC; this is probably due to the fact that acACS considers the protein secondary structure information. The sensitivity (Sn), Matthew’s correlation coefficient (MCC), and accuracy (Acc) of AAC were all higher than DC, because DC displays redundant or irrelevant features, so we used “F-score” to select the feature. As shown in Figure 4, the closer the color is to red, the higher the F-score of adjacent amino acid residue and the easier it is to distinguish. On the contrary, the closer the color is to blue, the harder it is to distinguish. It can be seen that DC has some redundant information; this redundant information will reduce the prediction success rate. Figure 5 showed the Acc of DC based on the incremental feature selection (IFS). The peak (the maximum accuracy) can be found in this curve, and it was 90.93% with 245D features. Figure 6 showed the comparison of DC with feature selection and non-feature selection; we can see that feature selection was successfully applied to remove the irrelevant and redundant features. The Sn, MCC, and Acc were improved remarkably; Acc increased from 86.67 to 90.93%, Sn increased from 38.24 to 60.29%, and the results indicate that feature selection was helpful to enhance the predictive performance. The predictive results of different combined features with SVM without SMOTE were displayed in Figure 7. From Figure 7 we can see the combined feature AAC+DC+acACS+PSSM-AC was better than other parameters. The accuracy (Acc) of combined feature AAC+DC+acACS+PSSM-AC was 95.20% with the jackknife test. This result indicates that the combined feature was powerful in the prediction of cell wall lytic enzymes.

**TABLE 1:** The predictive results of individual features with jackknife test by using SVM.

**Features**	**Sn (%)**	**Sp (%)**	**MCC**	**Acc (%)**
AAC	47.06	95.77	0.51	86.93
DC	38.24	97.39	0.48	86.67
PSSM-AC	72.06	99.67	0.81	94.40
acACS	57.35	93.81	0.55	87.20

### Comparison With Different Classifiers

In order to display the power of our predictive model, our predictive model [Support Vector Machine (SVM)], Random Forest (RF), K-Nearest Neighbors (KNN), and Naive Bayes (NB) were used to predict cell wall lytic enzymes. The predictive performance of SVM, RF, KNN, and NB were listed in Table 2. From Table 2, we can see the predictive performance of SVM, RF, KNN, and NB with SMOTE were superior to those without SMOTE. The Acc of SVM, RF, KNN, and NB increased by 3.99, 3.89, 9.37, and 10.38% when using SMOTE; the MCC of SVM, RF, KNN, and NB increased by 0.15, 0.37, 0.37, and 0.21 when using SMOTE. In addition, the Sn, Sp, MCC, and Acc of SVM reached 99.35%, 99.02%, 0.98, and 99.19% by using SMOTE. The experimental results show that SVM was useful for improving the predictive performance of cell wall lytic enzymes.

**TABLE 2:** The predictive results of combined feature AAC+DC+acACS+PSSM-AC by using different algorithms with and without SMOTE.

**Algorithms**	**SMOTE (N/Y)**	**Sn (%)**	**Sp (%)**	**MCC**	**Acc (%)**
SVM	N	75.00	99.67	0.83	95.20
RF		41.18	85.99	0.27	77.87
KNN		66.18	80.13	0.40	77.60
NB		86.76	66.78	0.42	70.40
SVM	Y	99.35	99.02	0.98	99.19
RF		85.99	77.52	0.64	81.76
KNN		100.00	73.94	0.77	86.97
NB		92.18	69.38	0.63	80.78

### Comparison With Existing Methods

To further investigate the effectiveness of our predictive model, we compared it with existing methods with the same dataset. The comparison results were listed in Table 3. From Table 3, we can see that the predictive results of cell wall lytic enzymes in our predictive model were better than those of the other methods. Furthermore, the Sn, Sp, MCC, and Acc in our predictive model reached 99.35%, 99.02%, 0.98, and 99.19%, which were 32.65%, 10.42%, 0.407, and 18.79% higher than the Ding et al. (2009) method, 22.88%, 5.86%, 0.302, and 7.89% higher than Lypred, and 14.05%, 1.32%, 0.135, and 3.69% higher than CWLy-SVM. These results indicate that our predictive model was superior to existing methods.

**TABLE 3:** The comparison of the predictive results between this paper and existing methods.

**Method**	**Sn (%)**	**Sp (%)**	**MCC**	**Acc (%)**
Ding et al.	66.70	88.60	0.573	80.40
Lypred	76.47	93.16	0.678	91.30
CWLy-SVM	85.30	97.70	0.845	95.50
Our predictive model	99.35	99.02	0.98	99.19

## Conclusion

With the rapid rise of antibiotic-resistant strains, cell wall lytic enzymes used to destroy bacteria is a viable alternative method to avoid the crisis of antimicrobial resistance. In this work, a reliable and effective computational method was developed to identify the cell wall lytic enzymes. This model was derived from the SVM machine learning algorithm; SMOTE was used to counter the imbalanced data classification problems, and the F-score algorithm was used to remove redundant or irrelevant features. A series of experiments demonstrated that the proposed method is powerful. This method has good capability for distinguishing lyases.

## Data Availability Statement

Publicly available datasets were analyzed in this study. This data can be found here: http://lin-group.cn/server/Lypred/data.html.

## Author Contributions

F-ML conceived the selection of feature parameters and performed the results analysis. X-YJ carried out the computation and wrote the manuscript. Both authors reviewed the manuscript.

## Conflict of Interest

The authors declare that the research was conducted in the absence of any commercial or financial relationships that could be construed as a potential conflict of interest.

## Footnotes

1 http://bioinf.cs.ucl.ac.uk/psipred/

2 http://202.207.14.87:8032/bioinformation/acACS/index.asp
